# TRiC/CCT chaperonins are essential for organ growth by interacting with insulin/TOR signaling in *Drosophila*

**DOI:** 10.1038/s41388-019-0754-1

**Published:** 2019-02-21

**Authors:** Ah-Ram Kim, Kwang-Wook Choi

**Affiliations:** 1 Department of Biological Sciences, Korea Advanced Institute of Science and Technology (KAIST), Daejeon, 305-701 Republic of Korea; 2 Present Address: Department of Genetics, Harvard Medical School, Boston, MA 02115 USA

**Keywords:** TOR signalling, Genetics, Experimental organisms, Genetics, Experimental organisms

## Abstract

Organ size is regulated by intercellular signaling for cell growth and proliferation. The TOR pathway mediates a key signaling mechanism for controlling cell size and number in organ growth. Chaperonin containing TCP-1 (CCT) is a complex that assists protein folding and function, but its role in animal development is largely unknown. Here we show that the CCT complex is required for organ growth by interacting with the TOR pathway in *Drosophila*. Reduction of *CCT4* results in growth defects by affecting both cell size and proliferation. Loss of *CCT4* causes preferential cell death anterior to the morphogenetic furrow in the eye disc and within wing pouch in the wing disc. Depletion of any CCT subunit in the eye disc results in headless phenotype. Overgrowth by active TOR signaling is suppressed by *CCT* RNAi. The CCT complex physically interacts with TOR signaling components including TOR, Rheb, and S6K. Loss of *CCT* leads to decreased phosphorylation of S6K and S6 while increasing phosphorylation of Akt. Insulin/TOR signaling is also necessary and sufficient for promoting CCT complex transcription. Our data provide evidence that the CCT complex regulates organ growth by directly interacting with the TOR signaling pathway.

## Introduction

Organ size is regulated by intercellular signaling for cell growth and proliferation during development. The insulin/TOR signaling pathway is a highly conserved mechanism for controlling cell and organismal growth [1, 2]. This pathway is activated in response to a variety of environmental cues such as nutrients, hypoxia, osmotic stress, and DNA damage [3]. Insulin signaling leads to activation of TOR kinase through signal transduction steps including PI3K, Akt, and Rheb. Activated TOR phosphorylates its targets S6K and 4E-BP to promote cell growth and also affects cell cycle regulators to increase proliferation. Dysregulation in insulin/TOR signaling causes systemic disorders, including cancer, diabetes, and aging [4].

*Drosophila* has been an excellent animal model for molecular genetic analysis of signaling pathways involved in organ growth in vivo. The insulin/TOR signaling pathway has been extensively characterized in developing imaginal discs, but relatively little is known about how formation and maintenance of the signaling components are regulated. In our attempt to identify new genes involved in organ growth, we found an RNAi line that causes severe growth defects in the eye and head. Intriguingly, the gene responsible for this RNAi phenotype encodes CCT4, a subunit of the chaperonin containing TCP-1 (CCT) complex. These findings raised the possibility that CCT plays a critical role in developing tissues.

CCT is a chaperonin complex that regulates protein conformation and function. It is a large double-ring complex with a central cavity. Each ring is composed of eight paralogous subunits (CCT1–CCT8) [5, 6]. CCT complex has been predicted to interact with approximately 10% of newly synthesized proteins, among which actin and tubulin are representative substrates of the complex [7–9]. Importantly, evidence is growing that CCT is involved in the development and progression of cancer by interacting with oncogenic factors that regulate tissue growth and apoptosis [10–14]. CCT can also assist the assembly of protein complexes such as von Hippel-Lindau (VHL) tumor suppressor protein complex, Gβγ dimer, Bardet–Biedl syndrome protein (BBSome) complex, and basal transcription factor TFIID [15–18]. In addition, CCT plays a role in the disassembly of mitotic checkpoint complexes (MCC) [19]. *CCT* loss-of-function mutations in *S. cerevisiae* cause lethality, indicating the essential role of CCT complex [20]. CCT complex is also involved in protein trafficking by interacting with intracellular chain of Orai1 membrane protein [21]. Several in vivo studies suggest that CCT might play roles in autophagy, cell division, cell migration, life span, and sarcomere assembly [22–26].

A recent proteomic analysis of InR/PI3K/Akt network in *Drosophila* has shown that approximately 10% of interacting partners with the network are dynamically phosphorylated in response to insulin stimulation and one of the known interacting partners is CCT8 [27]. In addition, it was reported that CCT2 is phosphorylated by p90 ribosomal S6 kinase (RSK) and p70 ribosomal S6 kinase (S6K) in mammalian cells in response to extracellular stimuli [28]. Phosphorylation of CCT subunits by extracellular signals suggests that folding activity of CCT complex might be tightly regulated by the insulin/TOR signaling pathway to accelerate protein synthesis. However, it is unknown whether loss of CCT complex function affects the growth signaling pathways in animal development and how the levels of CCT complex are regulated.

In this study, we show that the CCT complex is essential for *Drosophila* organ development by interacting with insulin/TOR signaling. Reduction of the CCT complex leads to decreases in phospho-S6K and phospho-S6 while increasing phospho-Akt. The CCT complex physically interacts with insulin/TOR signaling components like TOR, Rheb, and S6K. Growth defects by reduced CCT complex function can be partially suppressed by overexpressing *Cyclin E* (*CycE*). In addition, we found that insulin/TOR signaling regulates CCT complex levels at both mRNA and protein levels. To our knowledge, this study provides first in vivo evidence that the CCT complex is required for TOR signaling and that the transcription of CCT complex is regulated by insulin/TOR signaling.

## Results

### Identification of CCT4 as an essential gene for organ growth

**Fig. 1:**
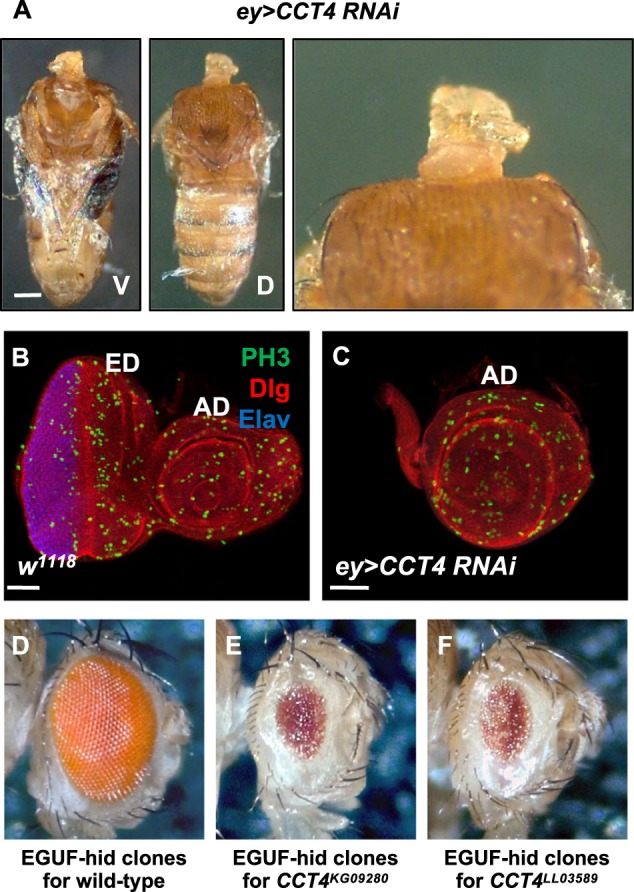
CCT4 is essential for eye and head development. **a** Adult body of *ey*>*CCT4 RNAi*. *ey*>*CCT4 RNAi* caused headless phenotype. V: ventral, D: dorsal. Scale bar, 500 μm. **b** Eye disc of wild-type (*w*^*1118*^). ED: eye disc, AD: antenna disc. Scale bars, 50 μm (**b**, **c**). **c** Eye disc of *ey*>*CCT4 RNAi*. *ey*>*CCT4 RNAi* led to complete loss of eye disc field (ED). **d** Wild-type EGUF-Hid clones. **e** EGUF-hid clones for *CCT4*^*KG09280*^. Whole-eye clones for *CCT4*^*KG09280*^ showed reduced eye size. **f** EGUF-hid clones for *CCT4*^*LL03589*^. Whole-eye clones for *CCT4*^*LL03589*^ showed reduced eye size. Anterior is to the right in **b**–**f**

CCT has been implicated in cell division and survival in eukaryotic cells [29, 30], but its role in tissue growth at organismal level remains largely unknown. Our preliminary finding of a CCT4 function in organ development led us to further investigate the effects of CCT4 loss in adult and developing organs. When *CCT4* was knocked down by RNAi (v106099) in eye discs by using *ey-Gal4* that drives GAL4 expression in eye and head primordia (hereafter *ey*>*CCT4 RNAi* for *ey-Gal4; UAS-CCT4 RNAi*), all progeny died during the late pupal stage (Fig. 1a). Similar results were obtained by an additional *CCT4* RNAi line (5525R-3). Dead flies in pupal cases had relatively intact thorax and abdomen but completely lacked the eye-head structures. We found that the eye-head field in larval eye discs targeted by *ey-Gal4* was lost (Fig. 1b, c), resulting in the headless phenotype and late pupal lethality. We also generated whole-eye mutant clones for *CCT4* using the *ey-Gal4 UAS-Flp*-*hid* (EGUF/hid) technique [31]. Eyes with EGUF-hid clones mainly consist of *CCT4* mutant cells because *CCT4*^+^ wild-type cells are selectively ablated by the expression of *hid* cell death gene. When EGUF-hid clones were generated by using two *CCT4* mutant alleles (*CCT4*^*KG09280*^ and *CCT4*^*LL03589*^), most of the animals with *CCT4* mutant clones died at pupal stages with headless phenotype. The size of *CCT4* mutant eyes of survived adult escapers was considerably reduced to about 20% of the wild-type control size (Fig. 1d–f), confirming that CCT4 is essential for eye development.

**Fig. 2:**
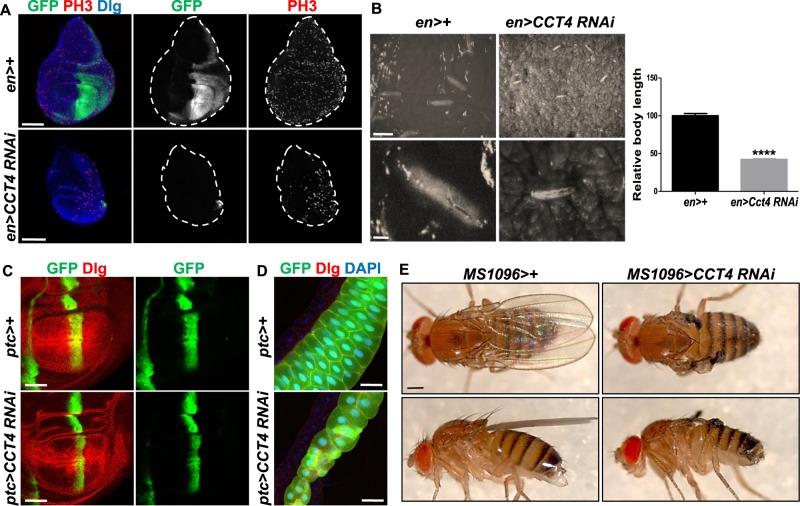
Knockdown of *CCT4* reduces body size with developmental delay. **a** Wing disc of *en*>+ and *en*>*CCT4 RNAi*. *en*>*CCT4 RNAi* led to loss of the *en-*expressing posterior compartment (GFP positive). Scale bar, 100 μm. **b** Relative body length of 5-day-old larvae of *en*>+ and *en*>*CCT4 RNAi*. *en*>*CCT4 RNAi* larvae showed shorter body length than *en*>+ larvae. *P*-value was calculated by using two-tailed *t*-test. ****P < 0.0001. Scale bars, 5 mm (top) and 1 mm (bottom). *n* *=* 5 (*en*>+); 5 (*en*>*CCT4 RNAi*). **c** Wing disc of *ptc*>+ and *ptc*>*CCT4 RNAi*. *ptc*>*CCT4 RNAi* showed reduced tissue size in *ptc-*expressing area (GFP positive). Scale bars, 100 μm. **d** Salivary gland of *ptc*>+ and *ptc*>*CCT4 RNAi*. *ptc*>*CCT4 RNAi* caused abnormal development of salivary gland. Scale bars, 50 μm. **e** Adult wing of *MS1096*>+ and *MS1096*>*CCT4 RNAi*. *MS1096*>*CCT4 RNAi* resulted in near complete loss of adult wing. Scale bars, 500 μm

To examine the roles of CCT4 in different organ development, we compared the effects of *engrailed* (*en*)>+ and *en*>*CCT4 RNAi* in wing discs*. CCT4* RNAi in the posterior wing disc under *en-Gal4* caused early pupal lethality. Wing discs from *en*>*CCT4 RNAi* showed almost complete loss of the posterior compartment marked by *en*>*GFP* (Fig. 2a). In comparison to control *en*>+, *CCT4*-depleted larvae showed considerable developmental delay during its growth up to the third-instar stage (Fig. 2b), eventually resulting in pupal lethality. *patched* (*ptc*)>*CCT4 RNAi* led to reduced tissue growth in the *ptc* domain along the anterior–posterior boundary of wing disc (Fig. 2c). Consistent with the expression of *ptc-Gal4* in the salivary gland, *CCT4* RNAi reduced the size of the gland compared with control (Fig. 2d). In addition, knockdown of *CCT4* in the wing pouch region of the developing wing disc under *MS1096-Gal4* led to loss of adult wing (Fig. 2e). Taken together, our data demonstrate that CCT4 plays important roles in development of multiple organs.

### Loss of the CCT complex leads to decreases in cell growth, division, and survival

**Fig. 3:**
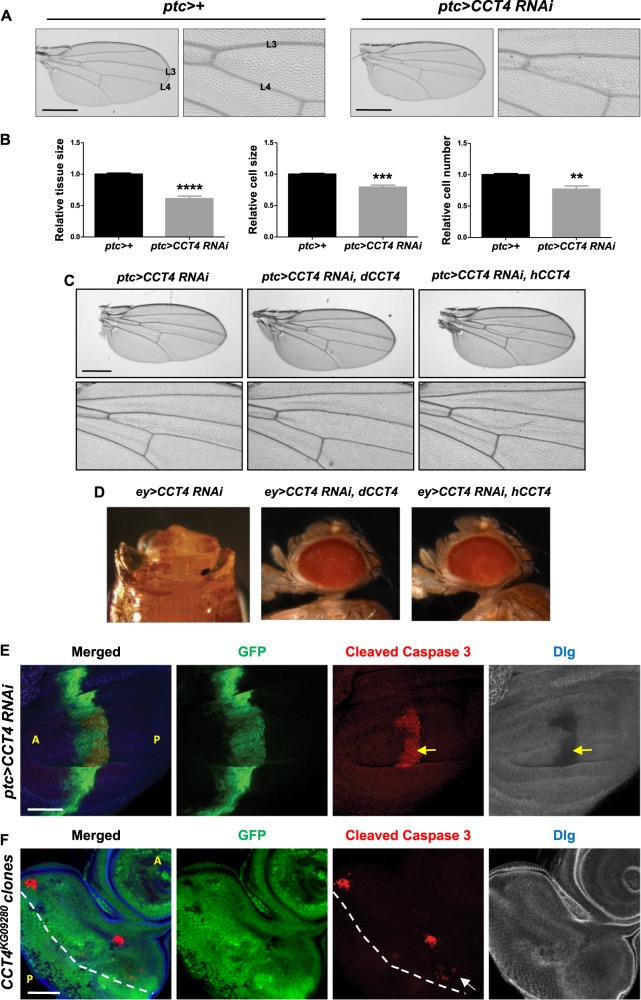
Reduced CCT4 affects cell number, size, and tissue-specific cell survival. **a** Adult wing of *ptc*>+ and *ptc*>*CCT4 RNAi*. *ptc*-expressing area of *ptc*>*CCT4 RNAi* was smaller than *ptc*>+. Scale bars, 400 μm. **b** Relative tissue size, cell size, and cell number in *ptc-*expressing area (between L3 and L4 veins) of *ptc*>+ and *ptc*>*CCT4 RNAi* adult wing. Tissue size, cell size, and cell number of *ptc*>*CCT4 RNAi* were reduced compared to *ptc*>+. **P < 0.01, ***P < 0.001, ****P < 0.0001. *n* *=* 5 (*ptc*>+); *n* = 5 (*ptc*>*CCT4 RNAi*). **c** *Drosophila CCT4* and human *CCT4* can rescue *ptc*>*CCT4 RNAi* phenotype. Adult wing of *ptc*>*CCT4 RNAi*. *CCT4* RNAi resulted in growth defect in the tissue between L3 and L4 veins. **d** *Drosophila CCT4* and human *CCT4* can rescue the headless phenotype of *ey*>*CCT4 RNAi*. **e** Wing disc of *ptc*>*CCT4 RNAi*. In *ptc*>*CCT4 RNAi* wing disc, ectopic cleaved Caspase-3 staining in *ptc-*expressing area (GFP positive) was shown in the basal region of wing pouch (white dashed line). Dlg is also strongly reduced in the region of cleaved Cas-3 staining (yellow arrow). **f** Ectopic cleaved Caspase-3 staining in eye discs with *CCT4*^*KG09280*^ mutant clones. Cleaved Caspase-3 staining was shown in *CCT4*^*KG09280*^ mutant clones (GFP-negative) and *CCT4*^*KG09280*^/+ heterozygous tissues (arrow). The morphogenetic furrow is indicated by the dashed line. A: anterior, P: posterior. Scale bars, 50 μm (**d**, **e**)

The data shown in Figs. 1 and 2 indicated that CCT4 is essential for tissue development. To check whether loss of CCT4 affects cell growth and/or division, we first compared the tissue size and measured the cell numbers in the *ptc* domain between L3 and L4 veins. As shown in Fig. 3a, *ptc*>*CCT4 RNAi* resulted in about 40% reduction in the region between L3 and L4 veins compared to *ptc*>+ control wing (Fig. 3a, b). Since each hair represents a single cell in the adult wing, we divided the tissue size by the hair numbers to estimate relative cell size. In *ptc*>*CCT4 RNAi*, both the relative cell size and cell number were reduced by 21% and 23% respectively (Fig. 3b), indicating that CCT4 is important for the regulation of both cell size and number. To exclude the possibility that the loss-of-function phenotype by *CCT4* RNAi is caused by off-target effects, we tested whether either fly CCT4 (dCCT4) or human CCT4 (hCCT4) can rescue the *ptc*>*CCT4 RNAi* phenotype. Growth defect resulting from *ptc*>*CCT4 RNAi* was rescued by overexpressing either *dCCT4* or *hCCT4* (Fig. 3c). More severe growth defects caused by *nub*>*CCT4 RNAi* or *ey*>*CCT RNAi* were also rescued by overexpressing either *dCCT4* or *hCCT4* (Supplementary Figure 1 and Fig. 3d). These results suggest that *CCT4* RNAi phenotypes are specific on-target effects.

To check whether loss of CCT4 affects cell survival during development, we immunostained wing discs with an anti-cleaved Caspase-3 (Cas-3) apoptosis marker and found that the level of cleaved Cas-3 was increased in *ptc*>*CCT4 RNAi* wing disc (Fig. 3e). A cell membrane marker Discs-large (Dlg) was reduced in the region of cleaved Cas-3 staining, consistent with cell death. Interestingly, cell death was mainly restricted to the posterior part of the *ptc* domain in the wing pouch. Furthermore, no cleaved Cas-3 staining was detected in the *ptc* domain outside the wing pouch. This suggests that CCT4 is required for cell survival in specific cell types.

We also examined the effects of *CCT* mutant clones in the eye disc. An eye disc can be divided into the anterior and the posterior region by the morphogenetic furrow [32]. The anterior region is composed of dividing cells, whereas the posterior region is mainly comprised of differentiating cells. Most of *CCT4* mutant clones were very small, indicating that mutant cells are defective in proliferation and/or cell survival. *CCT4* mutant clones in the anterior part of the eye disc showed positive-staining for cleaved Cas-3 (Fig. 3f and Supplementary Figure 2A). However, *CCT4* mutant clones in the posterior part of the eye disc did not show any detectable ectopic cell death. Interestingly, ectopic cell death was also found in antenna disc containing *CCT4*^*KG09280*^ mutant clones (indicated by an arrow in Supplementary Figure 2B). In addition, we checked whether RNAi knockdown of CCT complex subunits can cause ectopic cell death under *GMR-Gal4* driver that is expressed in the eye region posterior to the morphogenetic furrow. Consistent with the results from *CCT4* mutant clones, RNAi knockdown of any CCT complex subunit under *GMR-Gal4* did not cause ectopic cell death (Supplementary Figure 3A), resulting in normal adult eye morphology (Supplementary Figure 3B). These data indicate that the CCT complex is more critical for cell survival in dividing cells but not in differentiating cells.

In addition, we generated *CCT4* mutant clones at different time points and examined their effects. When clones were induced by heat-shock-inducible Flp (hs-Flp) 24 h before dissection, *CCT4*^*LL63589*^ mutant clones showed similar sizes compared with twin spots (Supplementary Figure 4A). However, when clones were induced 48 h before dissection, most mutant clones disappeared while twin spots grew larger (Supplementary Figure 4B). Hence, *CCT4* mutant cells can initially survive and divide up to 24 h but fail to survive afterwards, leading to the removal of mutant cells. The initial survival of *CCT4* mutant cells might be due to persistence of *CCT4* mRNA and protein. Nonetheless, these data suggest that *CCT4* is required for cell growth, division, and survival in cell- and tissue-dependent manners.

### All CCT subunits are required for the CCT complex function

**Fig. 4:**
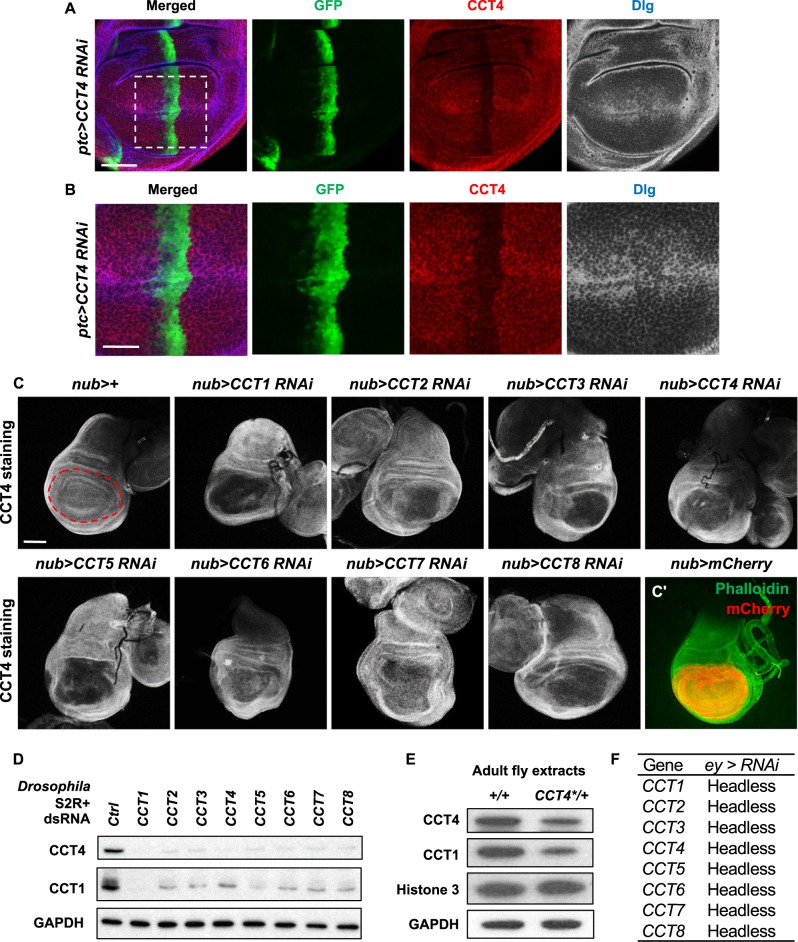
All CCT subunits are required for the CCT complex function. **a** CCT4 staining of *ptc*>*CCT4 RNAi* wing disc. *ptc*>*CCT4 RNAi* caused reduction in CCT4 staining in *ptc-*expressing area (GFP positive). Scale bar, 50 μm. **b** High magnification views of the box area in **a**. Scale bar, 25 μm. **c** CCT4 staining in wing discs with *CCT* subunit RNAi. *nub*>+ shows control wing pouch with even CCT staining. The *nub*-expressing area is marked by red dashed line. CCT4 staining in *nub-*expressing area was reduced when *CCT1*–*8* was knocked down. mCherry expression by *nub-Gal4* is shown in **c**′. Green staining indicates phalloidin. Scale bar, 100 μm. **d** Effects of *CCT* subunit RNAi on the CCT complex. CCT1 and CCT4 levels were decreased by reducing any of eight *CCT* subunits in S2R+ cells. GAPDH levels were used as a loading control. **e** Western blot of wild type and *CCT4*^*KG09280*^/+ adult extracts. The level of CCT4 was decreased in *CCT4*^*KG09280*^/+ extracts compared to wild-type control. Note that CCT1 was also reduced by the *CCT4* mutation. Histone 3 and GAPDH were used as loading controls. **f** All *CCT* subunit RNAi in *ey-Gal4* resulted in late pupal lethality with similar headless phenotypes

To examine the expression and stability of CCT4 proteins in developing tissues, we generated an anti-CCT4 antibody using a region of CCT protein (MBP-CCT4^385–524^). Specificity of the CCT4 antibody was confirmed by selective reduction of CCT4 protein level in the *ptc* domain of *ptc*>*CCT4 RNAi* wing disc (Fig. 4a, b). Reduction of CCT4 protein level was also confirmed in *CCT4*^*LL03589*^ wing mutant clones (Supplementary Figure 5). Since eight CCT subunits function together as a protein complex, it is possible that loss of one CCT subunit affects the stability of other subunit proteins. To test this possibility, we knocked down different *CCT* genes and checked the level of CCT4 protein. RNAi for *CCT1* to *CCT8* in the developing wing discs using *nub-Gal4* resulted in reduced CCT4 protein levels, suggesting that the level of CCT4 is affected by other CCT subunits (Fig. 4c). We also confirmed these results by western blot analysis. RNAi knockdown of *CCT1* to *CCT8* in S2R+ cells resulted in similar reduction of both CCT1 and CCT4 protein levels (Fig. 4d). Furthermore, we checked the effect of *CCT4* mutation on endogenous levels of CCT1 and CCT4 proteins. When adult fly extracts of wild-type control and *CCT4*^*KG09280*^/+ were examined on western blot, both CCT1 and CCT4 protein levels were reduced in *CCT4*^*KG09280*^/+ heterozygotes (Fig. 4e). Since loss of different CCT subunits resulted in reduction of CCT1 or CCT4 protein levels, it is possible that loss of any CCT subunit leads to similar loss-of-function phenotypes by disrupting the function of the CCT complex in vivo. As indicated in Fig. 4f, RNAi knockdown of any single CCT subunit under *ey-Gal4* driver caused pupal lethality with similar headless phenotypes. These data suggest that each subunit contributes to the function of the CCT complex.

### Genetic interaction between CCT complex and growth signaling pathways

**Fig. 5:**
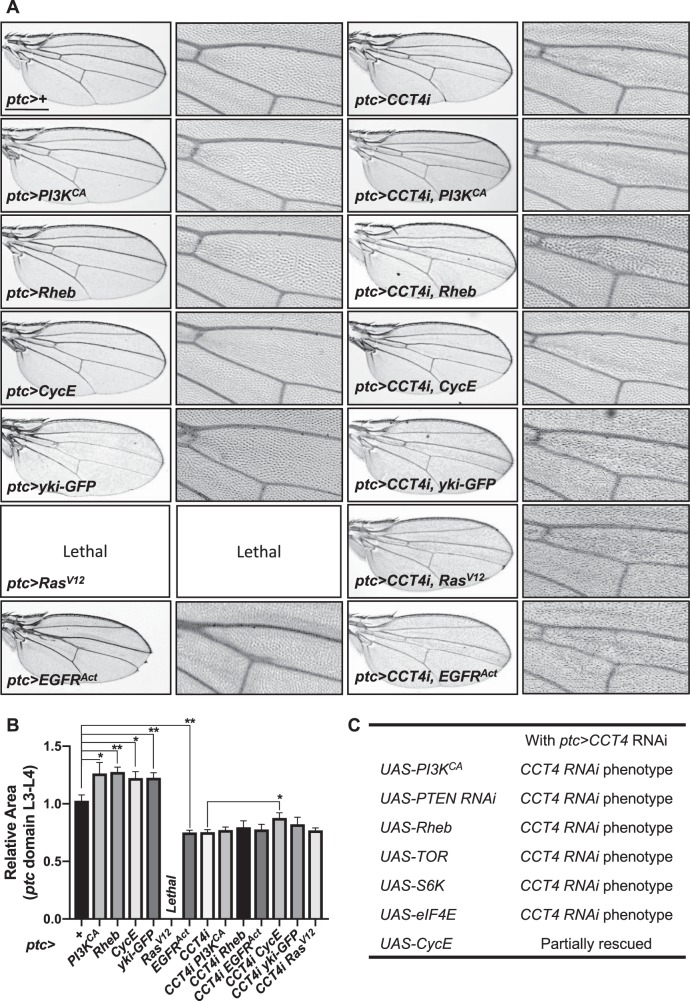
Genetic interaction between CCT complex and growth signaling pathways. **a** Adult wing images. *ptc*>+ is a control. Overexpression of *PI3K*^*CA*^, *Rheb*, *CycE*, and *yki-GFP* resulted in increase in *ptc*-expressing tissue. Overexpression of *Ras*^*V12*^ resulted in the embryonic lethality. Overexpression of *EGFR*^*Act*^ resulted in reduced wing size. *PI3K*^*CA*^, *Rheb*, *yki-GFP*, *ras*^*V12*^, or *EGFP*^*Act*^ overexpression was insufficient to rescue *ptc*>*CCT4i* phenotype. *CycE* overexpression partially rescued *ptc*>*CCT4 RNAi* phenotype. Scale bar, 400 μm. **b** Quantitative data on the size of the *ptc-*expressing domain shown in **a**. *P < 0.05, **P < 0.01. *n* = 3 (all genotypes). **c** Genetic interaction between *CCT4* and insulin/TOR signaling components

Defects in cell growth and division are one of the key characteristics in insulin/TOR signaling mutants. To test whether the CCT complex is associated with insulin/TOR signaling, we checked genetic interaction between *CCT4* and insulin/TOR signaling. As described earlier, *CCT4* RNAi under *ptc-Gal4* results in tissue reduction (Fig. 5a). When insulin/TOR signaling was activated by overexpressing *Pi3K*^*CA*^ and *Rheb* under *ptc-Gal4* driver, the wing tissue size between L3 and L4 veins was increased approximately 25%. However, *Pi3K*^*CA*^ or *Rheb* overexpression could not suppress the *CCT4* RNAi phenotype (Fig. 5a, b). These results suggest that the effects of *PI3K* or *Rheb* overexpression depend on CCT4 function. In S2R+ cells, *CCT4* RNAi did not affect the Rheb protein level (Fig. 8c), indicating that CCT4 function might be required to mediate the effects of *Rheb* overexpression instead of regulating the level of Rheb. In addition, *Rheb*^*2D1*^ loss-of-function mutation enhanced *ptc*>*CCT4 RNAi* phenotype (Supplementary Figure 6). Thus, *CCT4* RNAi shows genetic interaction with gain or reduction of Rheb function.

We also examined relationships between CCT and TOR downstream factors. Overexpression of *Tor*, *S6k*, and *eIF4E* could not rescue *ptc*>*CCT4 RNAi* phenotype (Fig. 5c). We then tested the effect of CycE since the CycE protein level is reduced in *Tor* mutants in *Drosophila* [33] and based on the fact that CycE is known as a folding substrate for the CCT chaperonin [34]. Interestingly, the reduced wing size resulting from *CCT4* RNAi was rescued by overexpressing *CycE* (Fig. 5a, b). The wing size suppression by *CycE* was partial but consistently observed in 100% of flies examined. CycE levels can also be regulated by other mechanisms such as Hippo and EGFR/Ras signaling pathways [35, 36]. Overexpression of *Yorkie* (*Yki*) by *ptc-Gal4* increased the *ptc* region in the wing but did not suppress the *CCT4* RNAi phenotype. Our data show that activated Ras (*Ras*^*V12*^) or EGFR (*EGFR*^*Act*^) overexpression by *ptc-Gal4* causes embryonic lethality or reduced wing, respectively (Fig. 5a). These effects might be due to non-autonomous cell death caused by activated Ras in imaginal discs [37]. Interestingly, *CCT4* RNAi flies expressing *Ras*^*V12*^ were viable, suggesting a role of CCT4 in Ras^V12^ function by an unknown mechanism. Nonetheless, activated Ras or EGFR did not affect the *CCT4* RNAi phenotype in the wing (Fig. 5a). Hence, CCT seems to be epistatic to not only TOR but also Yki and EGFR/Ras, implying possible roles of the CCT complex in multiple signaling pathways.

### CCT physically interacts with components of TOR signaling

**Fig. 6:**
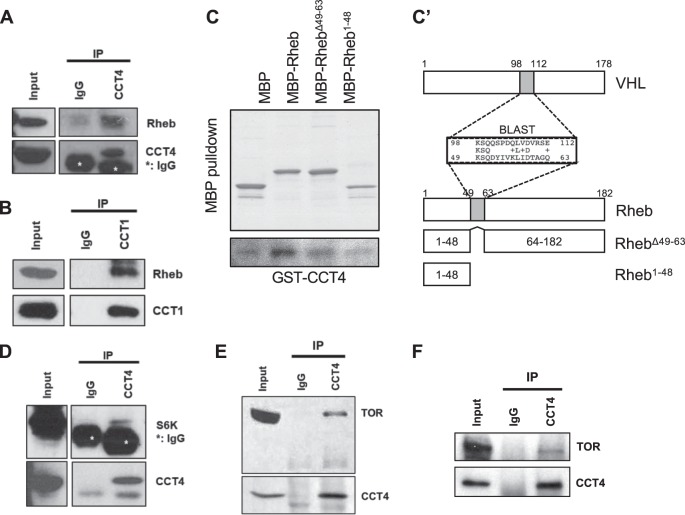
CCT complex physically interacts with TOR components. **a** Co-immunoprecipitation between endogenous CCT4 and Rheb using adult extracts. Asterisk indicates heavy chain IgG. **b** Co-immunoprecipitation between endogenous CCT1 and Rheb using adult extracts. **c** MBP-pulldown assay between MBP-Rheb and GST-CCT4. Rheb^Δ49–63^ and Rheb^1–48^ show little binding to CCT4 compared with the full-length Rheb. Sequence comparison in the related regions of VHL and Rheb is shown in (**c**′). Mutated Rheb constructs are also shown. **d** Co-immunoprecipitation between endogenous CCT4 and S6K using adult extracts. Asterisk indicates heavy chain IgG. **e** Co-immunoprecipitation between endogenous CCT4 and TOR using adult extracts. **f** Co-immunoprecipitation between endogenous CCT4 and Rheb using S2R+ cell extracts

Our data above suggest that the CCT complex genetically interacts with Rheb (Fig. 5 and Supplementary Figure 6). To further characterize this interaction, we tested physical interaction between CCT proteins and Rheb. We carried out co-immunoprecipitation using protein extracts from S2 cells transfected with Myc-CCT4 and Rheb-V5. As shown in Supplementary Figure 7A, Rheb-V5 was co-immunoprecipitated with Myc-CCT4. When either endogenous CCT1 or CCT4 protein was immunoprecipitated, endogenous Rheb was co-immunoprecipiated (Fig. 6a, b). Next, we performed MBP-pulldown assay to check whether CCT4 and Rheb directly interact. Pulldown assay showed that GST-CCT4 binds to MBP-Rheb (Fig. 6c and Supplementary Figure 7B), suggesting their direct interaction. Previously, it has been shown that a 55 amino acid domain of human VHL protein binds to CCT complex [15]. Sequence comparison indicated that Rheb has a 15 amino acid region that shows a weak similarity (33.3% identity/53.3% similarity) to the CCT complex-binding domain of VHL. Mutated Rheb deleted in this 15 amino acid region (Rheb^Δ49–63^) showed reduced binding to CCT4 (Fig. 6c–c′), suggesting that the deleted region is important for CCT complex binding as in the human VHL–CCT complex interaction. In addition, S6K was co-immunoprecipitated with CCT4 proteins in adult fly extracts (Fig. 6d), and MBP-pulldown assay indicated that S6K directly interacts with CCT4 (Supplementary Figure 7C). We found two regions of S6K that show low levels of similarity (26.7% identity/37.5% similarity) to the VHL domain for CCT complex binding. However, deletion of both regions did not affect the binding of S6K to CCT4 (Supplementary Figure 7C), suggesting that S6K interacts with CCT4 through a different domain(s). Finally, we also found that TOR protein was co-immunoprecipitated with CCT4 proteins in both adult fly extracts and S2R+ cell lysates (Fig. 6e, f). These data suggest that the CCT complex is physically associated with multiple TOR pathway components.

### Reduced CCT complex affects the level of phosphorylated S6k and Akt

**Fig. 7:**
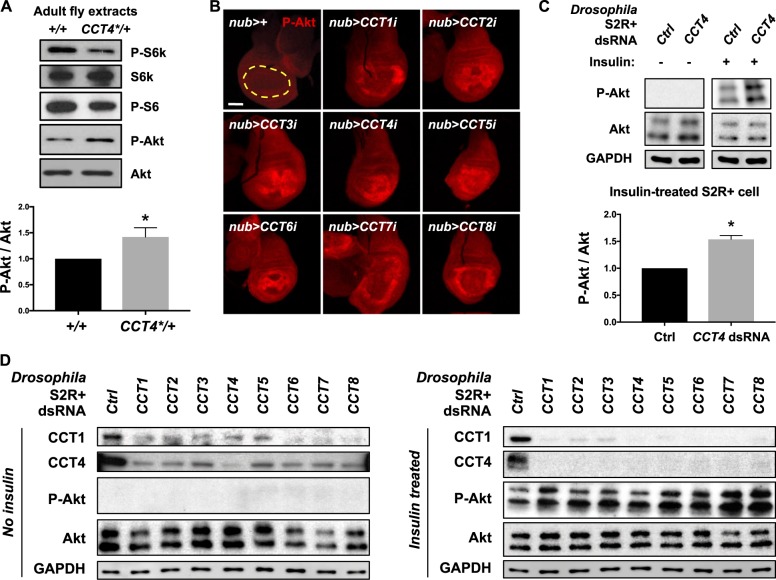
Reduced CCT complex affects the level of phosphorylated S6K and Akt. **a** Western blot of wild type and *CCT4*^*KG09280*^/+ adult extracts. Phospho-S6K and phospho-S6 levels were reduced in *CCT4*^*KG09280*^/+. Total S6K and Akt levels were not changed. P-Akt levels normalized to total Akt levels were shown in a bar graph. *P < 0.05. *n* = 3. **b** Increased phospho-Akt in *CCT*-knockdown wing discs. RNAi knockdown of each *CCT* gene using *nub-Gal4* increased P-Akt level compared to *nub*>+. *Nub-Gal4-*expressing area is marked by yellow dashed line. Scale bar, 100 μm. **c** *CCT4* RNAi increases the P-Akt level in insulin-treated S2R+ cells. P-Akt levels were increased in insulin-treated S2R+ cells. Insulin (25 µg/ml) was treated for 10 min. GAPDH levels were used as loading controls. P-Akt levels normalized to total Akt levels in insulin-treated S2R+ cells were shown as graph. *P *<* 0.05*. n* = 3. **d** The level of phospho-Akt in *CCT-*knockdown S2R+ cells. When insulin (25 µg/ml) was treated for 10 min, P-Akt levels were increased in each *CCT* RNAi cell lysate. GAPDH levels were used as loading controls

Since CCT complex and TOR signaling show genetic and physical interactions as shown above, we tested whether loss of *CCT4* affects insulin/TOR signaling. We checked the levels of phospho-S6K (P-S6K) and phospho-S6 (P-S6) in adult *CCT4*^*KG09280*^/+ heterozygous mutant extracts. In *CCT4*^*KG09280*^/+ heterozygotes, phosphorylation levels of S6K and S6 proteins were reduced while there was little change in the total S6K protein level (Fig. 7a), indicating that the CCT complex is required for activation of TOR kinase.

Akt/PKB signaling is a conserved pathway that integrates responses to growth factors, nutrients, metabolites, and stress. Akt/PKB signaling utilizes feedback mechanisms to regulate cell proliferation and cell size [38]. Activation of Akt/TOR signaling is also regulated by a negative feedback mechanism during normal *Drosophila* development. Phospho-Akt (P-Akt) was used as a marker for the negative feedback status in Akt/TOR signaling [39]. Since S6K is required for the regulation of the negative feedback in Akt/TOR signaling and the P-S6K level was reduced by *CCT4*^*KG0928*^ mutant heterozygotes, we checked whether reduction of CCT complex function results in any change in the P-Akt level. As shown in Fig. 7a, an aberrant increase in the P-Akt level was found in *CCT4*^*KG0928*^ mutant heterozygotes with no change in the total Akt protein level. To check whether the P-Akt level is increased by loss of CCT complex during development, we immunostained *CCT*-depleted wing discs with P-Akt antibody. When any of *CCT1–8* subunits was knocked down in wing discs, P-Akt level was increased (Fig. 7b). We also tested whether knockdown of the CCT complex increases the P-Akt level in S2R+ cells. Akt phosphorylation was detected only when the cells were treated with insulin. In insulin-treated cells, P-Akt level was significantly increased by *CCT4-*knockdown (Fig. 7c). Furthermore, P-Akt level was similarly elevated by depleting any of the eight CCT subunits in insulin-treated S2R+ cells (Fig. 7d). These data show that loss of CCT complex activity leads to decreased TOR signaling activity and increased P-Akt level.

### CCT complex levels are regulated by insulin/TOR signaling

**Fig. 8:**
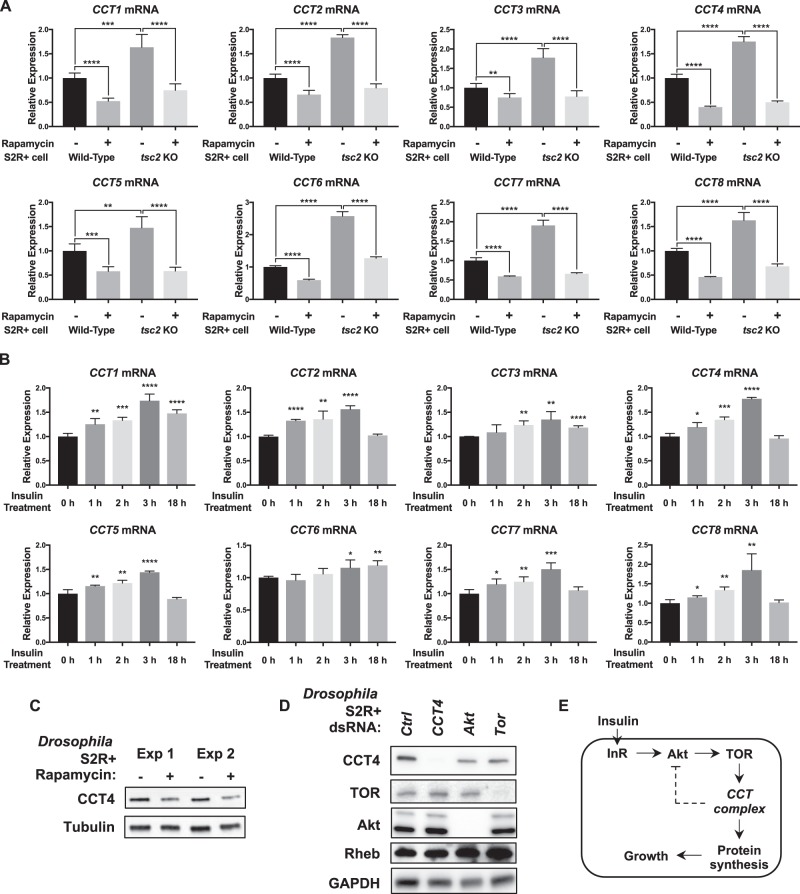
Insulin/TOR signaling activity affects the transcription levels of CCT complex. **a** Effects of rapamycin treatment on *CCT* mRNA levels in wild type and *tsc2* KO S2R+ cells. Rapamycin (20 nM) was treated for 24 h. All *CCT* mRNA expressions were decreased by rapamycin treatment. All *CCT* mRNA expressions were higher in *tsc2* KO cells compared to wild-type cells. Expression levels of *CCT* mRNA were normalized to *Rp49* levels. **P < 0.01, ***P < 0.001, ****P < 0.0001*. n* = 4. **b** Effects of insulin treatment on *CCT* mRNA levels in wild-type S2R+ cells. Insulin (25 µg/ml) was treated for the indicated time lengths. All *CCT* mRNA expressions were increased by 3 h insulin treatment. Some *CCT* mRNA levels were still elevated after 18 h insulin treatment. Expression levels of *CCT* mRNA were normalized to *Rp49* levels. *P < 0.05, **P < 0.01, ***P < 0.001, ****P < 0.0001*. n* = 4. **c** Rapamycin treatment decreases CCT4 protein levels. S2R+ cells were treated with rapamycin (20 nM) for 2 days. The same amounts of proteins were used for western blot. Two independent experiments were shown. Tubulin levels were used as loading controls. **d** Decrease in the CCT4 protein level of *Akt* or *Tor*-knockdown S2R+ cells. CCT4 protein levels were decreased when S2R+ cells were treated with *Akt* or *Tor* dsRNA. Protein levels of TOR, Akt, or Rheb were not changed by *CCT4* RNAi. GAPDH levels were used as loading controls. **e** A proposed scheme of interaction between CCT complex and insulin/TOR signaling. Transcription of *CCT* complex is regulated by insulin/TOR signaling. CCT complex function is required for the downstream process of TOR signaling to facilitate protein synthesis essential for cellular growth. After activation of insulin/TOR signaling, CCT complex is required for negative feedback regulation of Akt activity

Since insulin/TOR signaling increases protein synthesis upon activation, we hypothesized that CCT complex levels might also be regulated by TOR signaling. To test this hypothesis, we checked the level of all *CCT* mRNA in wild type and *tsc2* KO S2R+ cells with or without rapamycin treatment. As shown in Fig. 8a, all *CCT* mRNA levels were higher in *tsc2* KO cells compared to wild-type cells. Rapamycin treatment significantly reduced the levels of all *CCT* mRNA in both wild type and *tsc2* KO S2R+ cells, indicating that TOR signaling is required for transcription of all *CCT* subunits.

We also tested whether insulin treatment can enhance transcription of CCT complex genes. In wild-type S2R+ cells, all *CCT* mRNA levels were increased after insulin treatment (Fig. 8b), indicating that activation of insulin signaling is sufficient to increase CCT complex transcription. In addition, CCT4 protein levels were decreased when rapamycin was treated in S2R+ cells (Fig. 8c). The reduction of CCT4 protein levels was further confirmed in *Akt* or *Tor* RNAi-treated cells (Fig. 8d). These results suggest that insulin/TOR signaling regulates CCT complex expression at the transcriptional level, possibly affecting their protein levels (Fig. 8e).

## Discussion

In this study, we have shown that the CCT complex is essential for the regulation of cell growth, proliferation, and survival during *Drosophila* organ development by interacting with the insulin/TOR signaling pathway. We have demonstrated that CCT is critical for development of multiple organs including eye, wing, and salivary gland. Clonal analysis shows that *CCT4* mutant cells fail to proliferate and disappear by cell death. This indicates that CCT4 is cell-autonomously required for cell division and survival. Analyses of *CCT* RNAi effects in wings and salivary glands suggest that reduced CCT function not only affects cell number but also cell size, as commonly seen in tissues impaired by TOR pathway mutations.

Depletion of any *CCT* subunit by *ey-Gal4* resulted in loss of larval eye disc and the headless phenotype in adults (Figs. 1 and 4f), implying that CCT is required during early stages of eye-head development. However, the headless phenotype was not rescued by blocking cell death with *P35* overexpression (data not shown). Clonal analysis indicates that *CCT4* mutant cells can form small clones but these clones disappeared shortly by cell death (Fig. 3f and Supplementary Figure 2). Interestingly, cell death in *CCT4* mutant clones is restricted to the region anterior to the morphogenetic furrow of the eye disc. Selective knockdown of *CCT* subunits posterior to the furrow by using *GMR-Gal4* did not induce ectopic cell death, resulting in nearly normal eyes (Supplementary Figure 3). Because the anterior and the posterior regions of the eye disc are mostly proliferating and differentiating, respectively, CCT complex seems to be preferentially required for promotion of cell proliferation and survival of dividing cells prior to the onset of differentiation.

Our study on wing shows that *CCT4* RNAi by *en-Gal4* or *MS1096-Gal4* can ablate the entire wing tissue. As in the eye, we found cell or tissue-specific requirements of CCT complex function. For example, RNAi in the developing wing using *ptc-Gal4* leads to ectopic cell death in the AP boundary within the wing pouch but not the surrounding area (Fig. 3e). A wing disc consists of the wing pouch in the central (distal) region and surrounding (proximal) region for hinge and other thoracic tissues. Thus, CCT complex appears to be essential for survival of wing cells but not for other thoracic parts. These data suggest that the CCT complex is involved in organ growth as a tissue-specific regulator rather than a general factor for cell survival.

The CCT complex consists of eight related subunits. An intriguing question is whether all eight subunits are essential for the structure and function of the CCT complex. Several observations support that all subunits depend on each other for the stability and function of the complex. First, loss of other CCT subunits leads to reduction of the CCT1 and CCT4 protein levels (Fig. 4c, d). Moreover, knockdown of any *CCT* subunit in eye disc causes similar headless phenotype (Fig. 4f). A recent zebrafish study has also reported that mutants in any CCT subunit show similar defects likely affecting the stability of the CCT complex [25]. Therefore, it seems that all CCT subunits in *Drosophila* and zebrafish contribute to non-redundant functions for the CCT complex.

Earlier studies have suggested that the function of CCT can be modulated by TOR signaling. In mammalian cells, CCT2 is phosphorylated by S6 kinase upon growth factor treatment, and the CCT2 phosphorylation is important for cell proliferation [28] although the phosphorylation site (Ser-260) of mammalian CCT2 is not conserved in *Drosophila* CCT2. Insulin-dependent phosphorylation of CCT8 subunit has been observed in *Drosophila* cell culture [27], but its physiological significance is unknown. Our data in this study provide evidence for a link between CCT complex and insulin/TOR signaling. Reduced cell number and cell size caused by *CCT* mutations or RNAi are characteristic of insulin/TOR signaling mutants. Our genetic and biochemical data support a role of CCT complex in TOR signaling. Activating insulin/TOR signaling fails to induce overgrowth when *CCT4* is depleted (Fig. 5a). In addition, we also found that *Rheb*^*2D1*^ mutation enhanced *CCT4* RNAi phenotype (Supplementary Figure 6). These data suggest a functional relationship between CCT and insulin/TOR signaling. Furthermore, *CCT4* heterozygous mutation causes a reduction of P-S6K while the total level of S6K is unchanged (Fig. 7a). Thus, CCT is required to promote TOR-dependent S6K phosphorylation. It is also noteworthy that depletion of any *CCT* subunit leads to an increase in P-Akt level (Fig. 7b, d). Since S6K activity is associated with the negative feedback regulation of Akt activity, the decreased S6K activity by loss of CCT function may be responsible for the ectopic upregulation of Akt/TOR signaling.

Our results demonstrate that loss of CCT function results in severe growth defects, but these phenotypes cannot be suppressed by overexpression of TOR pathway factors such as Rheb and S6K. One possibility is that CCT might be necessary for the stability of overexpressed TOR components. However, this possibility may be unlikely because we have not noticed detectable changes in the level of several TOR components such as TOR, Akt, and Rheb by *CCT4* RNAi (Fig. 8d). Alternatively, overexpression of any one TOR substrate may be insufficient to suppress the *CCT* RNAi phenotypes because TOR kinase regulates multiple target proteins. It should be noticed that the phenotypes by *CCT* mutations or RNAi seem to be more severe than those of *Tor* null mutation (*Tor*^*ΔP*^) described earlier [33]. Development of *Tor* mutants is arrested at larval stage, and mutant clones in imaginal discs are similar in size to their twin spots at 48 h after induction. Afterwards, clones are eliminated probably due to cell competition with adjacent wild-type cells. *CCT4* mutant clones showed similar loss of mutant cells within 48 h after induction (Supplementary Figure 4), suggesting that *CCT* mutant phenotypes may be even more severe than that of *Tor* null mutation. Several possibilities can be considered. Firstly, different levels of maternal expression of *Tor* and *CCT* genes from heterozygous females may affect the severity of phenotypes of homozygous mutant offspring. Secondly, *CCT* mutations may cause pleiotrophic phenotypes because CCT complex can interact with a number of proteins in addition to TOR signaling components. According to our genetic interaction data, the activation of Yki or EGFR signaling alone fails to alter the *CCT* RNAi phenotype, suggesting that *CCT* is epistatic to Yki and EGFR as well as TOR. Since CCT complex are likely to be involved in multiple signaling pathways, activation of multiple signaling pathways might be needed to rescue *CCT4* RNAi phenotypes. More details about the relationships between the CCT complex and other signaling pathways need future studies.

Our data indicate that CCT proteins physically interact with multiple TOR signaling components. Thus, loss of CCT complex may result in more severe phenotypes than loss of a single TOR signaling factor. This possibility is consistent with our data that CCT4 physically interacts with at least three TOR factors, Rheb, S6K, and TOR kinase (Fig. 6). Several studies have suggested that CCT complex can provide a platform for organizing protein complexes and assist the complex formation [15–18]. The CCT complex might be required for the formation of an active TOR complex by physically interacting with multiple TOR factors.

Lastly, we found that transcription of CCT complex was reduced by rapamycin treatment or increased by *tsc2* KO or insulin treatment (Fig. 8a, b), suggesting that CCT complex is not only required but also regulated by TOR signaling. Remarkably, our data reveal that CCT complex function is tissue specific. It is an intriguing possibility that interactions between the CCT complex and TOR signaling proteins are modulated in cell or tissue-specific manner. It remains to be studied whether the functional relationship between CCT complex and TOR signaling shown in this study is conserved in vertebrate systems.

## Experimental procedures

### Fly genetics

All *Drosophila* strains were grown and maintained at 25 °C. *UAS-CCT1 RNAi* (5374R-2, NIG and v34070, VDRC), *UAS-CCT2 RNAi* (v108615, VDRC and 7033R-1, NIG), *UAS-CCT3 RNAi* (v36070, VDRC and 8977R-1, NIG), *UAS-CCT4 RNAi* (v106099, VDRC and 5525R-3, NIG), *UAS-CCT5 RNAi* (8439R-1, NIG and v109505, VDRC), *UAS-CCT6 RNAi* (v108596, VDRC and v23751, VDRC), *UAS-CCT7 RNAi* (v108585, VDRC and 8351R-3, NIG), *UAS-CCT8 RNAi* (v103905, VDRC and 8258R-2, NIG), *UAS-PI3K*^*CA*^ (8294, BDSC), *UAS-Rheb*, *UAS-pten RNAi* (25841, BDSC), *UAS-TOR* (7012, BDSC), *UAS-S6k* (6912, BDSC), *UAS-eIF-4E* (8650, BDSC), *UAS-CycE* [40], *UAS-EGFR*^*CA*^ (59843, BDSC), *UAS-Ras85D*^*V12*^ (4847, BDSC), *UAS-yki.GFP* (28815, BDSC), *Rheb*^*2D1*^*/TM6B* [41], *ey-Gal4*, *ptc-Gal4*, *en-Gal4*, *nub-Gal4*, *MS1096-Gal4* were used. For generation of *UAS-dCCT4* and *UAS-hCCT4*, coding cDNA sequences of *Drosophila CCT4* and human *CCT4* were cloned into pUAST attB vector using one-step SLIC method [42] and cloned vectors were injected into P2 (3L, 68A4) sites using site-specific Phi31-mediated insertion (GenetiVision) [43]. For generation of *CCT4* mutant clones, *FRT42D CCT4*^*KG09280*^*/CyO* (111690, Kyoto DGRC) or *FRT42D CCT4*^*LL03589*^*/CyO* (141081, Kyoto DGRC) was crossed with *hsflp; FRT42D ubiGFP*, and first-instar larvae were treated with heat shock for 60 min at 37 °C. For EGUF clones, *FRT42D GMR-hid l(2)CL-R*^*1*^*/CyO; ey-Gal4 UAS-flp* flies (5251, BDSC) were used for crossing with *FRT42D CCT*^*KG09280*^*/CyO* or *FRT42D CCT4*^*LL03589*^*/CyO*.

### Immunostaining

Third-instar larvae were dissected in ice-cold phosphate-buffered saline (PBS). Collected tissues were fixed at 4% paraformaldehyde in PBS for 15 min. After washing twice with PBS, fixed tissues were blocked in 5% normal goat serum/PBT (PBS + 0.3% Triton X-100) for 30 min at room temperature. Samples were incubated with primary antibodies in 5% NGS/PBT at 4 °C for overnight. Following antibodies were used: rabbit anti-CCT4 (1:2000) (this study), rabbit anti-Dlg (1:500) (Kyung-Ok Cho, KAIST), mouse anti-Dlg (1:100) (DSHB), sheep anti-GFP (1:100) (Bio-Rad, 4745-1051), rabbit anti-Cleaved Caspase-3 (Asp175) (1:100) (Cell Signaling, 9661), rabbit anti-phospho-Drosophila Akt (Ser505) (1:100) (Cell Signaling, 4054). After washing three times with PBT, secondary antibody conjugated with FITC (1:100), Cy3 (1:600), or Cy5 (1:500) (Alexa Fluor, Molecular Probes) were incubated for 1 h at room temperature. For Phalloidin staining, Alexa Fluor 488 Phalloidin (Molecular Probes A12379) was incubated for 1 h at room temperature. After washing three times with PBT, Vectashield (Vector Laboratories) antifade reagent was used for mounting prepared samples. A Carl Zeiss LSM710 confocal microscope was used to acquire fluorescent images.

### Immunoprecipitation

For co-immunoprecipitation using adult fly extracts, S2 cell extracts, and S2R+ cell extracts, 1 mg of protein lysates were used after lysis using IP buffer (20 mM HEPES (pH 7.4), 0.2 mM EDTA, 1.5 mM MgCl_2_, 1 mM DTT, 5% glycerol, 80 mM KCl, 0.2% NP-40, protease inhibitor cocktail (Roche), and phosphatase inhibitor cocktail (Roche)). The lysates were incubated with antibodies for overnight at 4 °C, and then 50 μl of SureBead Protein A (Bio-Rad) was added and incubated for 1 h at 4 °C. After washing in IP buffer four times, proteins were eluted in 2× sample buffer.

### Pulldown assay

For pulldown assay, plasmids for MBP-Rheb and GST-CCT4 were used to transform IPTG-inducible BL21 competent cells. For cloning partial Rheb or S6k constructs, corresponding DNA fragments were amplified by PCR and cloned into pMAL-c2 vector using In-Fusion cloning (Takara). Tagged proteins were purified using a standard method. Pulldown buffer (20 mM Tris pH 7.5, 150 mM NaCl, 0.5 mM EDTA, 10% glycerol, 0.1% Triton X-100, 1 mM DTT, and protease inhibitor cocktail) was used. For western blot analysis, rabbit anti-MBP antibody (1:5000, Santa Cruz sc-271524), rabbit anti-GST antibody (1:5000, Santa Cruz sc-138 hrp), and secondary anti-rabbit antibody conjugated with HRP (Jackson) were used for western blotting.

### Cell culture, reagents, RNA interference, and transfection

S2 cells, wild-type S2R+ cells, and *tsc2* KO S2R+ cells [44, 45] were grown in Schneider’s Drosophila Media (Thermo Fisher Scientific) supplemented with 10% FBS (Thermo Fisher Scientific) and penicillin–streptomycin. For insulin treatment, 25 µg/ml of insulin was treated for the indicated time lengths. For rapamycin (LC Laboratories) treatment, cells were incubated with 20 nM rapamycin for the indicated time lengths.

For RNAi experiments, PCR templates for dsRNA against *CCT1* through *CCT8* were prepared using primers designed by SnapDragon-dsRNA design (http://www.flyrnai.org/snapdragon). dsRNAs for *CCT1-8* were generated by PCR using MEGAscript T7 (Ambion) and purified using MEGAClear (Ambion). Thirty micrograms of dsRNA was treated in S2R+ cells in six-well plates for 3 days using bathing method [46].

For immunoprecipitation between Myc-CCT4 and Rheb-V5 proteins, coding sequences of CCT4 and Rheb were cloned into pAc5.1-V5/His (Invitrogen) with N-terminal Myc tag with and without C-terminal stop codon, respectively. The cloned constructs were transfected in S2 cells using Effectene reagent (Qiagen).

### Quantification of mRNA expression

Total RNA of S2R+ cells was extracted by TRIzol (Thermo Fisher Scientific). cDNAs were synthesized by iScript cDNA Synthesis Kit (Bio-Rad). CFX96 Real-Time System (Bio-Rad) and iQ SYBR green supermix (Bio-Rad) were used for quantitative PCR. All expression data were normalized to *Rp49*. Primers for CCT1-8 were designed using FlyPrimerBank [47]. The primer sequences are listed in Supplementary Information.

### Immunoblotting

For protein extraction from S2R+ cells, cell lysates were extracted in lysis buffer (50 mM TrisCl, pH 7.6, 150 mM NaCl, 1 mM EDTA, 1% Triton X-100) with protease inhibitor cocktail (Roche) and phosphatase inhibitor cocktail (Roche). For protein extraction from adult flies, one-day-old flies were collected, and proteins were extracted in lysis buffer with protease inhibitor cocktail (Roche) and phosphatase inhibitor cocktail (Roche). Protein concentrations of the cleared lysate after two times centrifugation (13,000 r.p.m.) at 4 °C were measured, and the same amount of proteins was used for western blot analysis.

For immunostaining of western blots, following antibodies were used: mouse anti-V5 (1:5000, Invitrogen R960-25), mouse anti-Myc (1:1000, Santa Cruz sc-47694), rabbit anti-CCT4 (1:20,000) (this study), rat anti-CCT1 antibody (1:1000, Abcam ab90357), rat anti-Rheb (1:500) [48], and mouse S6K (1:1000) [40], rabbit phospho-S6K (1:1000, Cell signaling 9209), rabbit phospho-S6 (1:5000) [49], rabbit anti-Histone 3 (1:10,000, Millipore 05-928), rabbit anti-GAPDH (1:5000, GeneTex 100118), rabbit anti-Akt (1:1000, Cell Signaling 4691), rabbit anti-phospho-Akt (1:1000, Cell Signaling 4054), mouse anti-Tubulin (1:5000, Sigma T5168), guinea pig anti-TOR (1:2000) [50], rat anti-TOR (1:2000) [51].

### Statistical analysis

Statistical analyses were performed by GraphPad Prism. Statistical significance was determined by unpaired two-tailed Student’s *t*-test. All experiments were performed at least three times. *P*-values of <0.05 were considered as statistically significant. All data represent the mean ± s.e.m. (standard error of the means).

## Supplementary information

Supplementary Figure legend and Table.

Supplemental Figure 1

Supplemental Figure 2

Supplemental Figure 3

Supplemental Figure 4

Supplemental Figure 5

Supplemental Figure 6

Supplemental Figure 7
