# Supplementary material for: TRiC/CCT chaperonins are essential for organ growth by interacting with insulin/TOR signaling in Drosophila
Source: Oncogene. 2019 Feb 21;38(24):4739–54. doi: 10.1038/s41388-019-0754-1 (PMC6756063; doi:10.1038/s41388-019-0754-1)
Supplement: Supplementary file 1 — Supplementary Figure legend and Table. [file 41388_2019_754_MOESM1_ESM.docx]

**Supplementary Information**

**Supplementary Figure 1. *CCT4* RNAi phenotypes can be rescued by fly *CCT4* and human *CCT4*.** (A) *nub>CCT4 RNAi* caused a wing growth defect. The defect of *nub>CCT4 RNAi* is rescued by expressing fly *CCT4* (B) or human *CCT4* (C).

**Supplementary Figure 2. Cell death in *CCT4* mutant clones preferentially occurs in anterior eye disc.** (A-B) *CCT4^KG09280^* mutant clones result in cell death anterior to the morphogenetic furrow marked by white dashed line. Mutant clones are marked by the absence of GFP. (B) High magnification views of the box area in (A). Note that cell death occurs occasionally in *CCT4^KG09280^* heterozygote antenna tissues. (C) Wild-type control eye disc. No cell death is observed. Anterior is to the right. ED: Eye disc, AD: Antenna disc.

**Supplementary Figure 3. Normal larval and adult eyes of *CCT* subunit RNAi under GMR-Gal4.** (A) *GMR-Gal4* is expressed posterior to the morphogenetic furrow indicated by yellow dashed line. (B) *GMR>CCT1-8 RNAi* flies show normal morphology. Anterior is to the right.

**Supplementary Figure 4. CCT4 is essential for cell survival.** Heat-shock timing is shown in the experimental scheme. (A) 24 h *CCT4^LL63589^* mutant clones showed similar size to twin spots marked by bright GFP signal. Mutant clones are marked by the absence of GFP. (B) 48 h *CCT4^LL63589^* mutant clones are almost eliminated.

**Supplementary Figure 5. Quality check of CCT4 antibody.** CCT4 staining is reduced in *CCT4^LL63589^* mutant clones marked by the absence of GFP (indicated by arrows).

**Supplementary Figure 6. Genetic interaction between *CCT4* and *Rheb*.** Loss-of-function phenotype of *CCT4* is enhanced by *Rheb* mutation. *ptc>CCT4 RNAi* caused a reduction in the tissue size between L3 and L4 veins. Addition of *Rheb^2D1^* mutation to *ptc>CCT4 RNAi* genotype resulted in severe wing defects compared to *ptc>CCT4 RNAi*. Scale bars, 400 μm

**Supplementary Figure 7. Physical interaction between CCT complex and Rheb or S6K.** (A) Co-immunoprecipitation between Myc-CCT4 and Rheb-V5 in S2 cells. Rheb-V5 was immunoprecipitated by anti-V5. Myc-CCT4 was co-immunoprecipitated. (B) Pull-down assay between CCT4 and Rheb. GST-CCT4 and MBP-Rheb physically interact. Bead and GST were used as negative controls. Ponceau staining showed GST and GST-CCT4 proteins. (C) Pull-down assay between CCT4 and S6K. MBP-S6K and MBP-S6K^1-403^ physically interact. Sequence comparison in the related regions of VHL and S6K is shown in (C’). Partial S6K constructs are also shown.

**Supplementary Table 1. Primers used for CCT complex RNAi.**

| Primer Nme | Primer Sequence |
| --- | --- |
| CCT1 F | TAATACGACTCACTATAGGGAGATGGGTGTCCAGGTCTTG |
| CCT1 R | TAATACGACTCACTATAGGGGCCTTAGTGCCCTTGATGAG |
| CCT2 F | TAATACGACTCACTATAGGGGATTCAGCCCAACTGGTTTC |
| CCT2 R | TAATACGACTCACTATAGGGCAGCACTCATCCTCGAATCA |
| CCT3 F | TAATACGACTCACTATAGGGGCAGATCGAGGAGGAGTTTG |
| CCT3 R | TAATACGACTCACTATAGGGTATGATCTCTAGGGCGTGGG |
| CCT4 F | TAATACGACTCACTATAGGGCAAGATCAAGTGCATGGTCG |
| CCT4 R | TAATACGACTCACTATAGGGGTTCTCAGCCAGAGTGGAGG |
| CCT5 F | TAATACGACTCACTATAGGGATCCAAGGACAAGATGCTGG |
| CCT5 R | TAATACGACTCACTATAGGGGACTCCACCACGTTATGGCT |
| CCT6 F | TAATACGACTCACTATAGGGATTGCCAGTGCCGATAAGAC |
| CCT6 R | TAATACGACTCACTATAGGGTGCAGTCCTCAACGAAAGTG |
| CCT7 F | TAATACGACTCACTATAGGGGCGAGTTCCTTAAGCAGGTG |
| CCT7 R | TAATACGACTCACTATAGGGTCGTGGATCTTAGCCAGCTT |
| CCT8 F | TAATACGACTCACTATAGGGACCAAGTCTGAGGTGGTTCG |
| CCT8 R | TAATACGACTCACTATAGGGCAGTAGCCCAACTCCTCCTG |

**Supplementary Table 2. Primers used in quantitative PCR.**

| Primer Name | Primer Sequence |
| --- | --- |
| CCT1 F | GACCGCTCCCGTTGATGAG |
| CCT1 R | CTCTGCGTCAGCACCAATGA |
| CCT2 F | ACTGGGAGACTCGTTCCTGG |
| CCT2 R | ATGGGCGTGTTGGCAATCA |
| CCT3 F | TCCACCCCACTGTTATCATTCG |
| CCT3 R | CTGGATGCTAAGCTGGGACTG |
| CCT4 F | CAGAAGGGTCTGCATCCCAC |
| CCT4 R | GAGATCCACAGAGGTTTCCTTG |
| CCT5 F | AGCTGATGGAGGTTGACCAC |
| CCT5 R | GAATGGGATGAATGCCCCTG |
| CCT6 F | AGATGCAGCACAAATCCGATAC |
| CCT6 R | CCAAACGCTTCGGCATGTC |
| CCT7 F | TGCCAAGTCACAAGACGCC |
| CCT7 R | CACGAAGGGTTTCACCTGCTT |
| CCT8 F | ATGGCTTTATCCGTTCCCAAG |
| CCT8 R | CATGGTCTGTGCAAACTCCTT |
| Rp49 F | ATCGGTTACGGATCGAACAA |
| Rp49 R | GACAATCTCCTTGCGCTTCT |
