# Supplementary figures and images for: TRiC/CCT chaperonins are essential for organ growth by interacting with insulin/TOR signaling in Drosophila
Source: Oncogene. 2019 Feb 21;38(24):4739–54. doi: 10.1038/s41388-019-0754-1 (PMC6756063; doi:10.1038/s41388-019-0754-1)

# Supplementary Figure 1

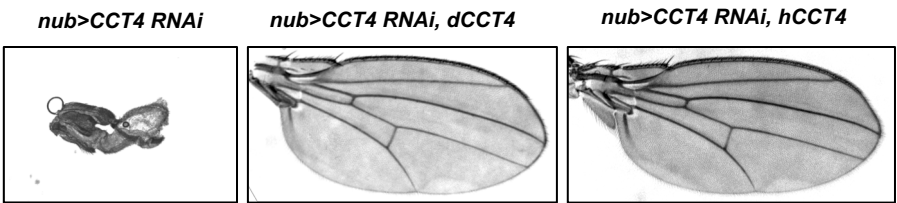

Supplement: Supplementary file 2 — Supplemental Figure 1 [file 41388_2019_754_MOESM2_ESM.pdf]

## Supplementary Figure 2

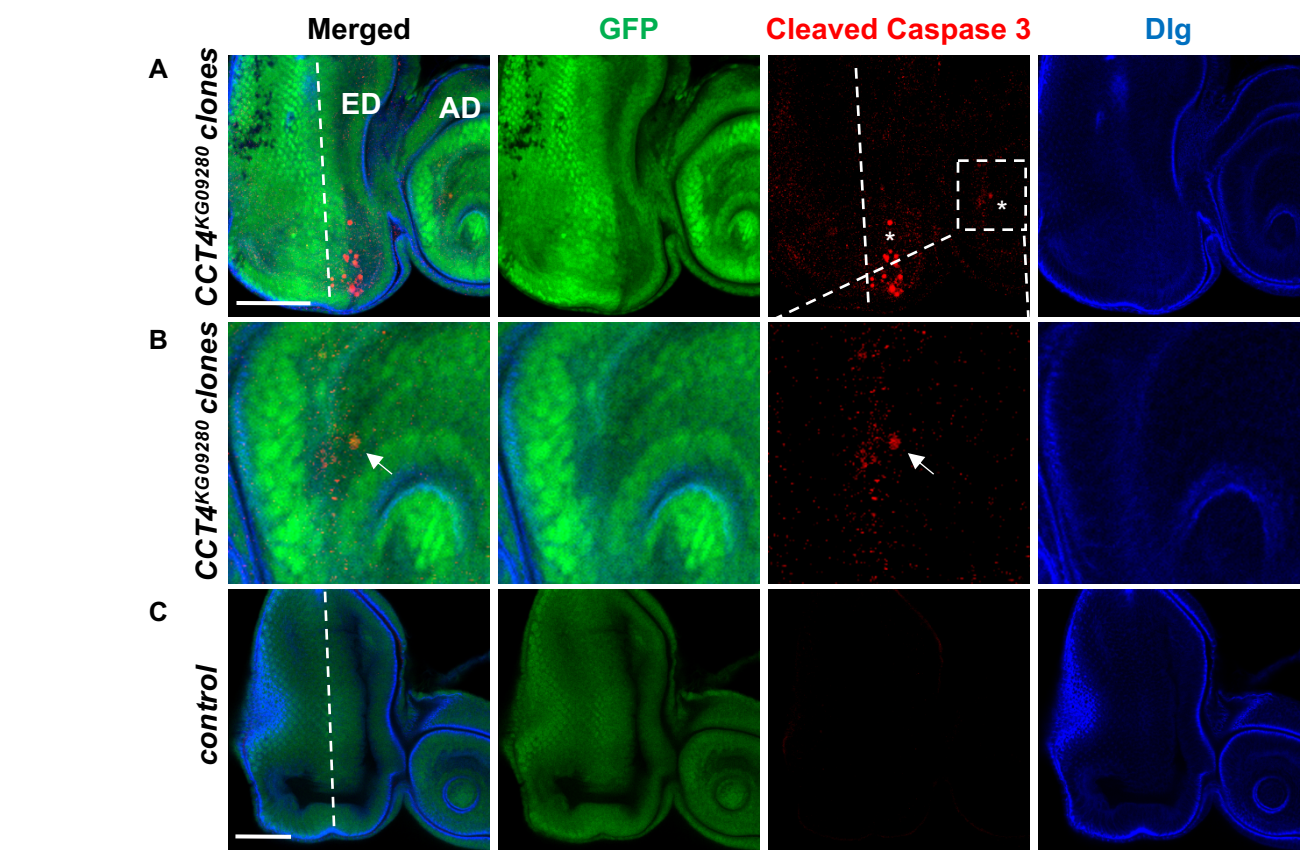

Supplement: Supplementary file 3 — Supplemental Figure 2 [file 41388_2019_754_MOESM3_ESM.pdf]

# Supplementary Figure 3

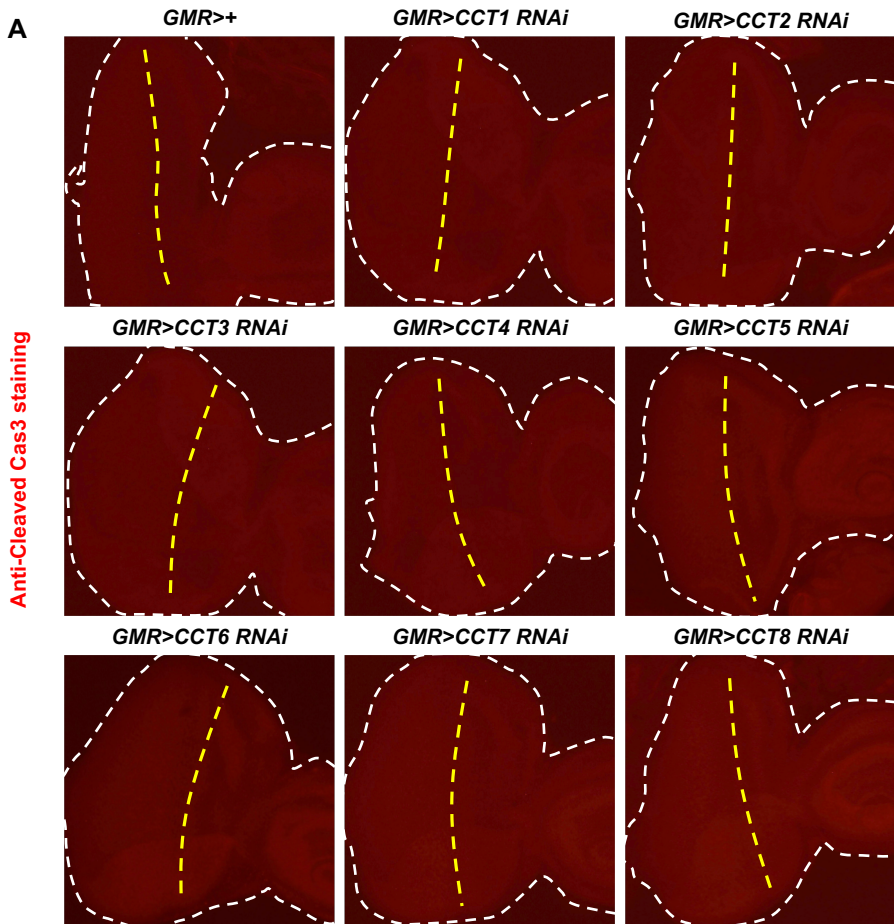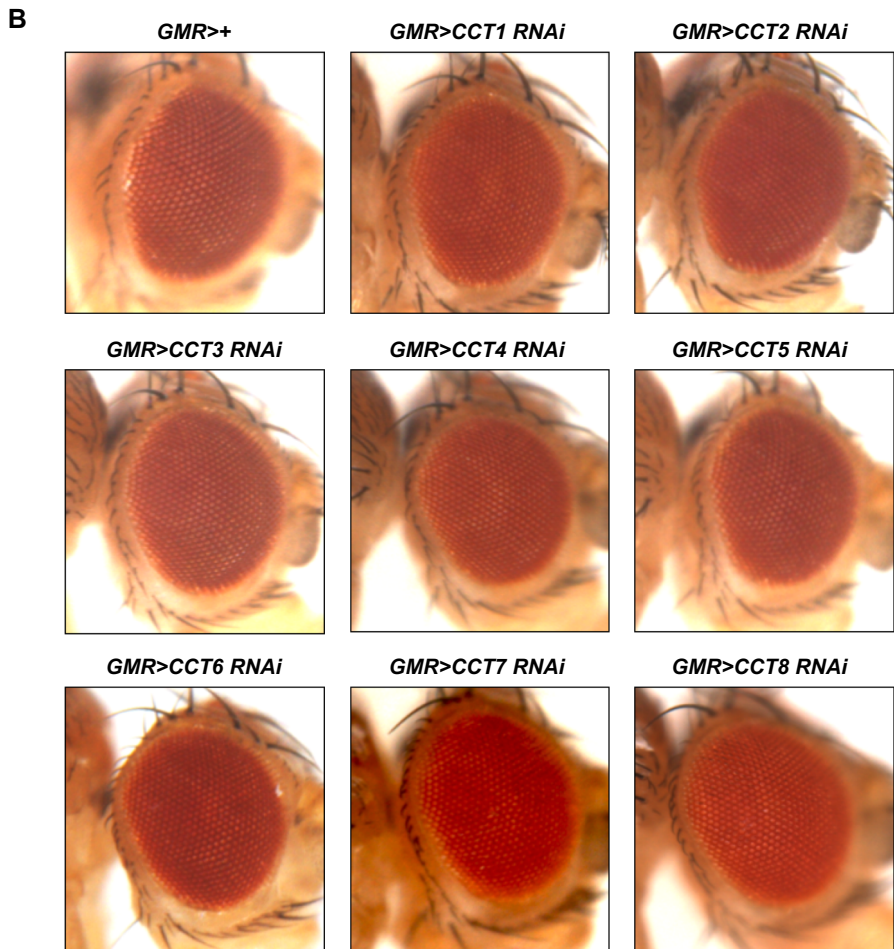

Supplement: Supplementary file 4 — Supplemental Figure 3 [file 41388_2019_754_MOESM4_ESM.pdf]

Supplementary Figure 4

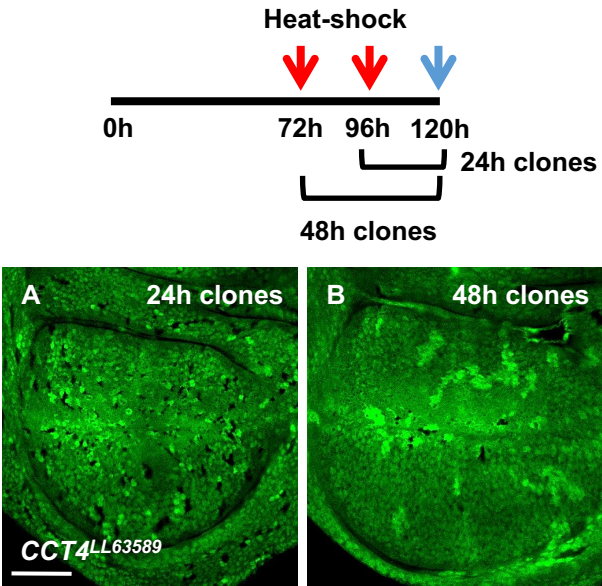

Supplement: Supplementary file 5 — Supplemental Figure 4 [file 41388_2019_754_MOESM5_ESM.pdf]

Supplementary Figure 5

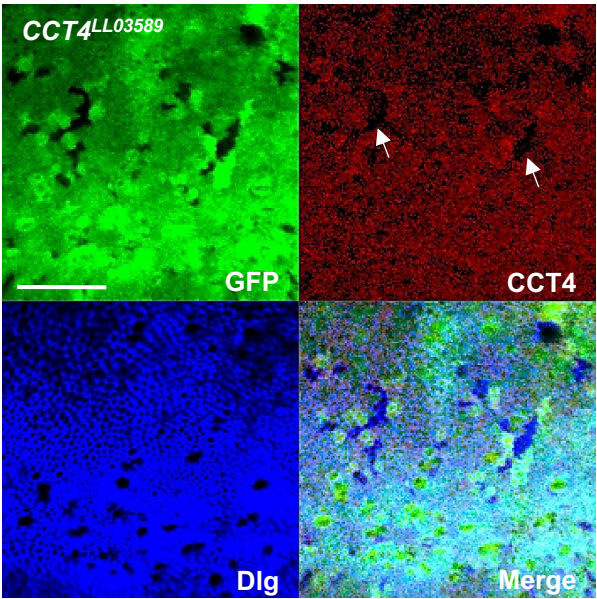

Supplement: Supplementary file 6 — Supplemental Figure 5 [file 41388_2019_754_MOESM6_ESM.pdf]

Supplementary Figure 6

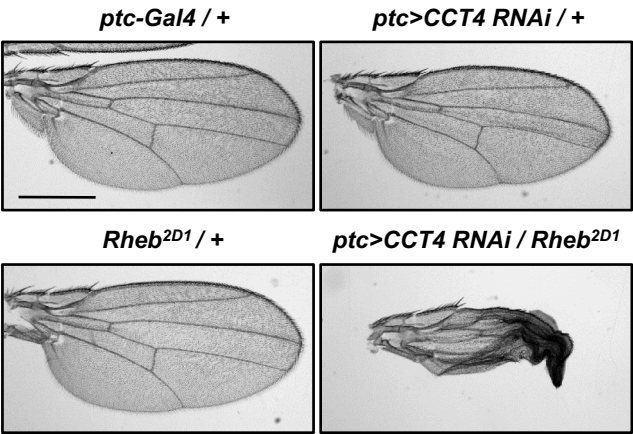

Supplement: Supplementary file 7 — Supplemental Figure 6 [file 41388_2019_754_MOESM7_ESM.pdf]

Supplementary Figure 7

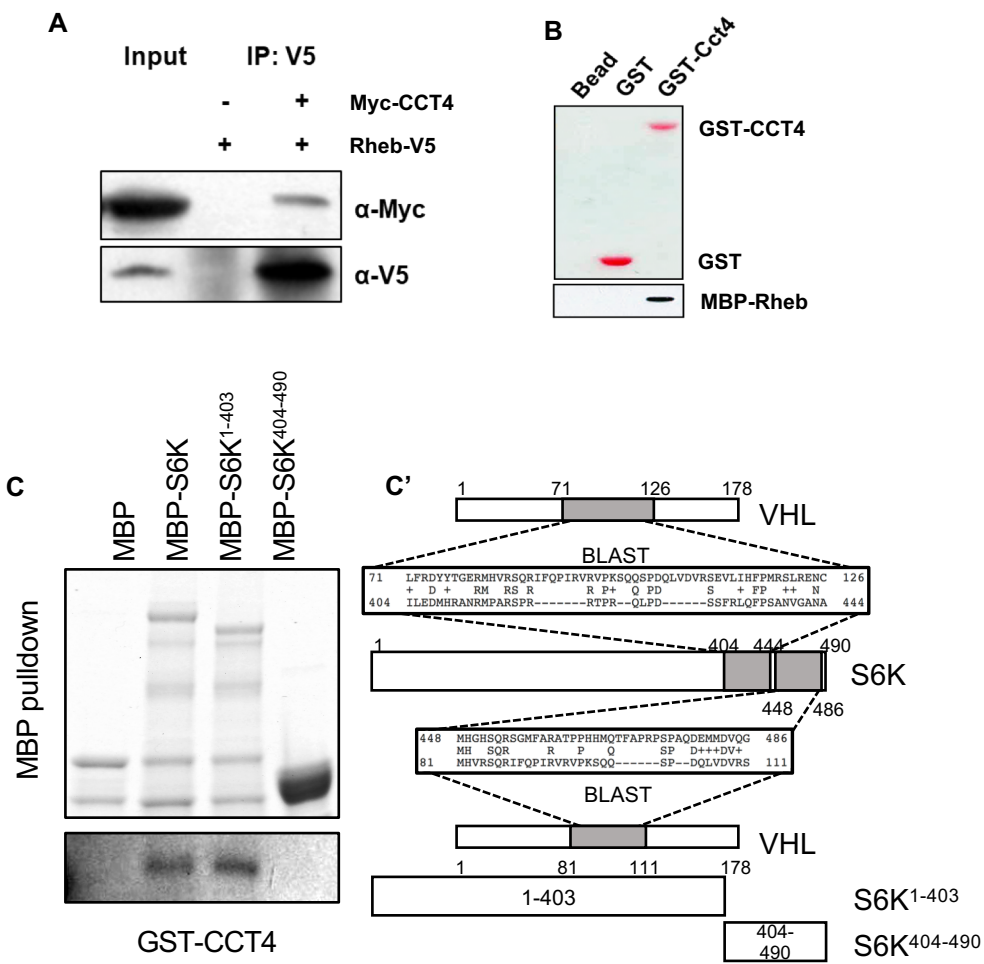

Supplement: Supplementary file 8 — Supplemental Figure 7 [file 41388_2019_754_MOESM8_ESM.pdf]
